# Characterization of Mechanical Allodynia and Skin Innervation in a Mouse Model of Type-2 Diabetes Induced by Cafeteria-Style Diet and Low-Doses of Streptozotocin

**DOI:** 10.3389/fphar.2020.628438

**Published:** 2021-02-03

**Authors:** Gabriela Castañeda-Corral, Norma B. Velázquez-Salazar, Arisai Martínez-Martínez, Juanita N. Taboada-Serrano, Pablo N. Núñez-Aragón, Laura González-Palomares, Rosa Issel Acosta-González, Vera L. Petricevich, Juan José Acevedo-Fernández, Sergio Montes, Juan Miguel Jiménez-Andrade

**Affiliations:** ^1^ Facultad de Medicina, Universidad Autónoma del Estado de Morelos, Cuernavaca, México; ^2^ Unidad Académica Multidisciplinaria Reynosa Aztlán, Universidad Autónoma de Tamaulipas, Reynosa, México; ^3^ Departamento de Neuroquímica, Instituto Nacional de Neurología y Neurocirugía “Dr. Manuel Velasco Suárez”, Ciudad de México México.

**Keywords:** painful distal polyneuropathy, type 2 diabetes mellitus, skin hypersensitivity, mechanical allodynia, skin nerve fibers, cafeteria diet.

## Abstract

**Background: **Painful distal symmetrical polyneuropathy (DPN) is a frequent complication of type-2 diabetes mellitus (T2DM) that commonly presents as neuropathic pain and loss of skin nerve fibers. However, there are limited therapies to effectively treat DPN and many of the current animal models of T2DM-induced DPN do not appear to mirror the human disease. Thus, we validated a DPN mouse model induced by a cafeteria-style diet plus low-doses of streptozotocin (STZ).

**Methods: **Female C57BL/6J mice were fed either standard (STD) diet or obesogenic cafeteria (CAF) diet for 32 weeks, starting at 8 weeks old. Eight weeks after starting diets, CAF or STD mice received either four low-doses of STZ or vehicle. Changes in body weight, blood glucose and insulin levels, as well as oral glucose- and insulin-tolerance tests (OGTT and ITT) were determined. The development of mechanical hypersensitivity of the hindpaws was determined using von Frey filaments. Moreover, the effect of the most common neuropathic pain drugs was evaluated on T2DM-induced mechanical allodynia. Finally, the density of PGP -9.5^+^ (a pan-neuronal marker) axons in the *epidermis* from the hindpaw glabrous skin was quantified.

**Results: **At 22–24 weeks after STZ injections, CAF + STZ mice had significantly higher glucose and insulin levels compared to CAF + VEH, STD + STZ, and STD + VEH mice, and developed glucose tolerance and insulin resistance. Skin mechanical sensitivity was detected as early as 12 weeks post-STZ injections and it was significantly attenuated by intraperitoneal acute treatment with amitriptyline, gabapentin, tramadol, duloxetine, or carbamazepine but not by diclofenac. The density of PGP-9.5^+^ nerve fibers was reduced in CAF + STZ mice compared to other groups.

**Conclusion: **This reverse translational study provides a painful DPN mouse model which may help in developing a better understanding of the factors that generate and maintain neuropathic pain and denervation of skin under T2DM and to identify mechanism-based new treatments.

## Introduction

Diabetes mellitus (DM) is a highly prevalent heterogeneous group of metabolic disorders characterized by hyperglycemia due to an absolute or relative deficit in insulin production or action. Currently, DM represents a severe public health problem in the world; 463 million people worldwide were estimated to have DM in 2019, and is expected that this number will increase to 700 million by 2045 (IDF, 2019). The most common type of diabetes is type-2 diabetes mellitus (T2DM) accounting for 90–95% of DM patients (ADA, 2019). T2DM results mainly from low physical activity and a high caloric diet leading to obesity (West and Kalbfleisch, 1971; Kolb and Martin, 2017). Obesity-induced T2DM is characterized mainly by chronic hyperglycemia and insulin resistance in peripheral tissues.

Among the T2DM long-term complications, diabetic neuropathies are one of the most common chronic complications, and distal symmetrical polyneuropathy (DPN) is the most prevalent type of diabetic neuropathy, with 30–50% of T2DM patients developing DPN (Candrilli et al., 2007). Although a large number of patients with DPN may be asymptomatic, approximately a quarter of people with T2DM suffer from painful DPN as a result of damage to or dysfunction of the sensory nerve fibers, along with a loss of the nerve fiber axons within the skin (Abbott et al., 2011). The affected patients with painful DPN often describe unpleasant sensory symptoms, burning pain, shooting pains down the legs; lancinating, contact pain often with day-time clothes and bedclothes (allodynia); pain when walking; sensations of heat or cold in the feet; persistent achy feeling in the feet and cramp-like sensations in the legs (Tesfaye, 2003). Although there has been significant progress in the understanding of the mechanisms underlying the development and maintenance of T2DM-induced painful DPN, there is still a limited repertoire of efficacious therapies to successfully treat it.

Preclinical models have been highly valuable tools to investigate the pathophysiology of T2DM-induced painful DPN. Current T2DM animal models are mainly based on genetic, dietary, chemical, surgical approaches, or a combination of chemicals administration with diet alterations (Srinivasan and Ramarao, 2007; Badole et al., 2013; Stenkamp-Strahm et al., 2015). However, genetic models might be relatively expensive and uneasily accessible for some investigators (Islam and Wilson, 2012). Furthermore, it has been pointed out that some of these preclinical models of T2DM do not mimic the pattern of the DPN development and progress that occurs in humans (Sullivan et al., 2008). Animal models resembling the human diabetic state have recently been developed; a miniature swine model of T2DM induced by a cafeteria-style diet (CAF) followed by streptozotocin (STZ), effectively mimics the natural history of the disease in humans, with diabetes and insulin resistance following a period of obesity (Coelho et al., 2018). The CAF diet consists of highly palatable and energy-dense foods which are very common in Western society (Sclafani and Springer, 1976; Sampey et al., 2011). The CAF diet along with the administration of STZ in Göttingen minipigs resulted in hyperphagia, ketonuria, and hyperglycemia (Coelho et al., 2018).

While this model of T2DM consisting of CAF followed by multiple low-doses of STZ has been characterized from a metabolic perspective in minipigs (Coelho et al., 2018), to our knowledge, there is no study that has adapted and characterized this model of T2DM in rodents to study the pathogenesis of painful DPN. Thus, we aimed to perform a reverse translational study to characterize the T2DM-induced long-term neuropathic pain-like behaviors, the effect of different analgesics on these behavioral pain-like responses, and the loss of nerve fiber axons within the glabrous skin of the mouse hindpaw.

## Materials and Methods

### Animals

Female C57BL/6J mice, aged initially six weeks old, were obtained from our breeding facilities. They were weight-matched and housed in groups of 4, in a temperature and humidity-controlled room (21 ± 2°C) under a 12:12 light/dark cycle (lights on at 07:00 h), with free access to food and tap water. All experimental procedures were performed following the Mexican Official Norm of Animal Care and Handling (NOM-062-ZOO-1999, 1999), the international guidelines on ethical standards for investigation in animals, and the international guidelines for the study of pain (Zimmermann, 1983). Moreover, the protocol was approved by the Institutional Animal Care and Use Committee of the Facultad de Medicina, Universidad Autónoma del Estado de Morelos. Efforts were made to minimize the number of animals used.

### Drugs

The following drugs/chemicals were used: STZ (lot number WXBC8740V), amitriptyline hydrochloride (lot number LRAA9172), duloxetine hydrochloride (lot number LRAB0283), carbamazepine (lot number MKCF7846), and gabapentin (lot number LRAA5714) were purchased from Sigma-Aldrich (St. Louis, MO). NPH insulin (lot number S19V698) and Tramadol hydrochloride parenteral solution were obtained from AMSA (Mexico City, Mexico) and Pisa Biotec (Guadalajara, Mexico), respectively. Diclofenac was donated by Senosiain S.A de C.V (Mexico City, Mexico). All drugs were dissolved in 0.9% sterile saline solution and prepared the same day they were tested.

### Type-2 Diabetes Model Induced by CAF-Style Diet and Low-Doses of STZ

**FIGURE 1:**
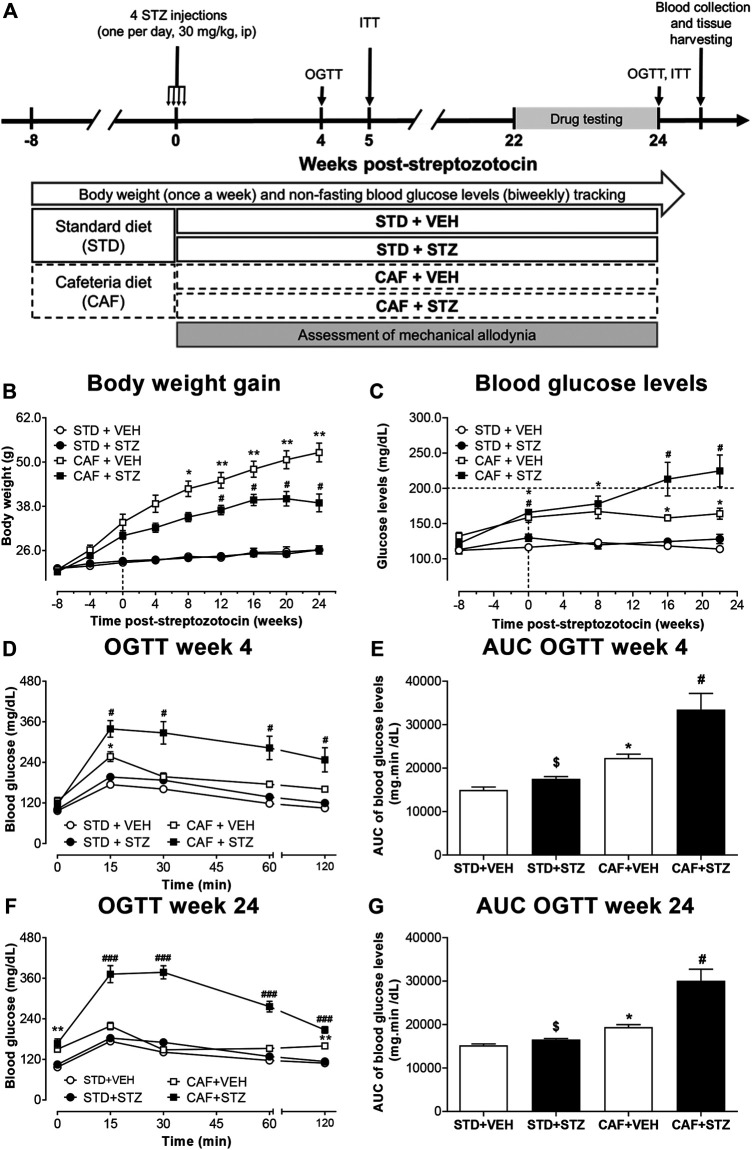
Outline of the experimental design **(A)** and changes in body weight **(B)**, blood glucose levels **(C)**, and glucose tolerance **(D–G)** in mice. Female C57BL/6J mice at 8 weeks-old were fed either cafeteria (CAF) or standard (STD) diet and injected with vehicle (VEH) or streptozotocin (STZ). Eight weeks later, CAF or STD mice were administered either with four low-doses of STZ (30 mg/kg, i.p., one per day) or vehicle. Values are means ± SEM (n = 10). In **(B)** *p < 0.05, **p < 0.01 for CAF [F_(1,36)_ = 109.5]. *, #*p* < 0.05 for the interaction CAF × STZ [F_(1,36)_ = 7.94]. In **(C)** *p < 0.05 for CAF [F_(1,36)_ = 74.11] #*p* < 0.05 for the interaction CAF × STZ × Time [F_(1,36)_ = 3.10]. In **(D)** *p < 0.05 for CAF [F_(1,36)_ = 33.84], #*p* < 0.05 for the interaction CAF × STZ × Time [F_(1,36)_ = 6.82]. In **(F)** *p < 0.05 for CAF [F_(1,36)_ = 150.6], ###*p* < 0.001 for the interaction CAF × STZ × Time [F_(1,36)_ = 22.16]. Notably, the interaction CAF × STZ showed an important effect on the variable (F = 48.8, *p* < 0.05). In **(E)** *p < 0.05 for CAF [F_(1,36)_ = 35.12], $ (*p* < 0.05) for STZ [F_(1,36)_ = 12.17], # (*p* < 0.05) for the interaction CAF × STZ [F_(1,36)_ = 4.75]. In **(G)** *p < 0.05 for CAF [F_(1,36)_ = 40.65], $ (*p* < 0.05) for STZ [F_(1,36)_ = 18.78], # (*p* < 0.05) for the interaction CAF × STZ [F_(1,36)_ = 11.73]. Data in **(B–F)** were analyzed by Repeated-Measures Three-Way ANOVA, followed by Bonferroni’s. Data in **(E)** and **(G)** were analyzed by Two-Way ANOVA. OGTT: oral glucose tolerance test.

After a two-week acclimation period, mice were randomly assigned to one of the two following dietary groups: 1) standard diet (STD) or 2) CAF diet. The STD diet provided an average of 19.49 Kcal/day, from which 10.7% is fat, 63% are carbohydrates and 26.1% is protein. On another hand, the CAF-diet used in this study was adapted from a CAF diet previously described by Leffa and coworkers (Leffa et al., 2015). This CAF-diet is rich in fat and carbohydrates (fat 36.5%, carbohydrates 42.2%, and protein 22.3%) and on average provides approximately 40.27 Kcal/day to each mouse. It consists of a choice of highly palatable, unhealthy human foods with known energy content and soft drinks such as orange-flavored and cola. Animals could select and freely consume the CAF items which were given in excess and daily replaced with fresh food (weekly menus and total energy value are outlined in Supplementary Table S1). To induce partial insulin deficiency after 8 weeks of feeding, the mice were subdivided into the following four groups: 1) STD + VEH, 2) STD + STZ, 3) CAF + VEH, and 4) CAF + STZ. Groups 1 and 3 were injected during four consecutive days with distilled water (0.1 ml/10 g body weight (BW), i.p.; vehicle), while groups 2 and 4 received four consecutive injections (i.p., one per day) of STZ at 30 mg/kg of BW (freshly dissolved in distilled water) (Zhang et al., 2008). After the last vehicle- or STZ-injection, all groups continually received their respective CAF or STD diets for the rest of the experiment. During the entire experimental period (32 weeks), the animals were weighed once a week (Figure 1A). Finally, at the 24th-week post-streptozotocin, the body mass index (BMI) was calculated as the ratio between body weight and square surface area according to the formula previously reported by Gargiulo et al. (Gargiulo et al., 2014). In all the experiments the number of experimental units per assay was 8–10. In this study a total of 62 mice were used as follows: STD + VEH (n = 10); STD + STZ (n = 10); CAF + VEH (n = 14); and CAF + STZ (n = 28). The number of animals from the CAF + STZ group was increased since they were used for assessing the analgesic drugs. In addition, from the CAF + STZ group, two mice were euthanized at week 18 post-STZ injections because they had lost more than 25% baseline BW and showed blood glucose levels above 400 mg/dL. Also, two other mice from this group were excluded because they had blood glucose levels below 200 mg/dL and normal glucose tolerance. Thus, the success rate of the model was higher than 80%.

### Blood Glucose Levels, Oral Glucose Tolerance Test, and Insulin Tolerance Test

Non-fasting blood glucose levels were monitored biweekly during the entire experimental period. Oral glucose tolerance test (OGTT) and insulin tolerance test (ITT) were performed at 4 and 24 weeks, and 5 and 24 weeks post-STZ, respectively (Figure 1A). In week 24 post-STZ, ITT was performed 4 days after the OGTT. In all cases, glucose quantification was performed in blood samples obtained from the tail vein blood using the calibrated glucometer system Accu-Chek Performa (Roche Diagnostic, Germany). For OGTT, mice fasted for 12 h received a bolus of aqueous glucose solution (2 g/kg) that was delivered into the stomach by a gavage probe (20-gauge, 38 mm long curved, with a 21/4 mm ball end). Blood glucose quantification was performed at 0 (basal), 15, 30, 60, and 120 min after the glucose load (Nagy and Einwallner, 2018). For ITT, mice with a 2 hours-fasting period were intraperitoneally injected with NPH insulin, which was dissolved in saline at a dose of 0.75 UI/kg BW (Nagy and Einwallner, 2018), and blood glucose levels were measured at the same time points as in the OGTT. For both, OGTT and ITT, the area under the curve (AUC) values for plasma glucose were calculated using the trapezoidal rule. It is important to mention that from the group CAF + STZ only mice that showed glucose levels above 200 mg/dL between 16 and 24 weeks post-STZ injections were used to perform OGTT (at 24 weeks), ITT (at 24 weeks), drug evaluation (weeks 22–24) and immunohistochemistry (24 weeks). Mice used to measure insulin or for immunohistochemistry did not receive any analgesic drug.

### Insulin Levels and Sensitivity

Insulin levels were measured by using the ELISA method in blood samples obtained 24 weeks post-STZ injections (Figure 1A). Blood samples were collected around 4:00–6:00 PM, by cardiac puncture and allowed to clot for 30 min at room temperature. Then, samples were centrifuged at 3,000 rpm/4°C for 15 min to separate the serum. The supernatant was aliquoted and frozen at −70°C until further processing. Insulin was measured by using a mouse insulin ELISA kit (Millipore, Cat. # EZRMI-13K) according to the manufacture’s specifications. The assay range for insulin was 0.1–10 ng/ml, and intraassay precision CV% < 10% and inter-assay precision CV% < 12%. All measurements were performed in duplicate and the mean of two measurements was considered. Insulin sensitivity was assessed by using the homeostasis model assessment-2 (HOMA2) index using an online-based calculator on the Diabetes Trials Unit of the University of Oxford website (https://www.dtu.ox.ac.uk/homacalculator) (Avtanski et al., 2019). To perform this, glucose levels were measured around 7:00–8:00 AM after a 12-h fasting period.

### Quantitative Assessment of Mechanical Allodynia

Mechanical allodynia, as a feature of painful DPN, was determined using von Frey filaments (Stoelting, Wood Dale, IL, United States). Briefly, 30 min before the behavioral evaluation, the mice were placed in individual acrylic boxes with a wire mesh grid to allow them to acclimatize to their surroundings. Then, the filaments were presented perpendicular to the mid-plantar surface of the left hind limb with enough force to cause slight buckling against the paw and held for 3–5 s. A positive response was noted if the paw was sharply withdrawn, flinching, or licking immediately. Nociceptive response for mechanical sensitivity was expressed as the 50% paw withdrawal threshold (g), and was calculated using the up-down method as described in Chaplan et al. (Chaplan et al., 1994). 50% paw withdrawal thresholds were assessed in all animals at 0, 4, 8, 12, 14, 16, 17, 18, 19, 20, 21, 22, 23, and 24 weeks after the last STZ injection. In all behavioral experiments, the investigator performing the evaluation was blinded to the experimental condition of the mice.

### Drug Testing

The antiallodynic effect of drugs, used as the first or second line of treatment for neuropathic pain, was evaluated when CAF + STZ mice displayed established allodynia (50% withdrawal thresholds ≤ 0.4 g; observed between 22 and 24 weeks post-STZ injection). To decrease the number of mice used for this aim, this experiment was performed in a total of 16 CAF + STZ mice using a crossover design (drug-vehicle-drug-vehicle). To do this the 50% withdrawal threshold was measured to confirm mechanical allodynia. Next, the 16 mice were numbered and then sorted into two groups (A and B, n = 8 each) by randomization using the GraphPad Prism software version 8 (GraphPad Software, Inc.). Posteriorly, mice in group (A) were administered with one of the analgesic drugs and the animals in group (B) received saline and then the anti-allodynic effect was assessed by measuring the paw withdrawal threshold at the multiple time-points indicated after the administration of each drug. After the experiment was finished, the animals rested for a washout period of at least seven elimination drug-half lives to ensure that 99% of the drug had been eliminated, and then, animals that were used as controls in the previous assay (B) were treated with the analgesic drug and the other group (A) served as control receiving saline. This procedure was repeated until complete the evaluation of the six analgesic drugs. Drugs and doses were chosen based on previous studies where they were effective in reducing neuropathic pain. The order in which the drugs were evaluated was gabapentin (100 mg/kg) (Wagner et al., 2014), tramadol (30 mg/kg) (Codd et al., 2008), duloxetine (30 mg/kg) (Kremer et al., 2018), carbamazepine (100 mg/kg) (Kiguchi et al., 2004), diclofenac (50 mg/kg, negative control) (Grim et al., 2014), and amitriptyline (30 mg/kg) (Kremer et al., 2018).

### Tissue Harvesting and Immunohistochemistry

Animals used for immunohistochemistry (IHC) were sacrificed 24 weeks post-STZ injections. Mice were deeply anesthetized with a mixture of ketamine and xylazine (100/10 mg/kg) followed by transcardiac perfusion first with phosphate-buffered saline (PBS, 0.1 M, pH 7.4, 4°C) and followed by 4% paraformaldehyde in PBS. Immediately after perfusion, the visceral adipose tissue, defined as the sum of epididymal, perirenal, mesenteric, and inguinal subcutaneous adipose tissue, was removed and weighed (Loredo-Pérez et al., 2016; Bagchi and MacDougald, 2019). Moreover, both posterior hindpaws were harvested, post-fixed for 24 h in the same fixative, and stored in PBS. Then, the skin of the hindpaw was cryoprotected in 30% sucrose solution at 4°C for further processing by IHC. Serial frozen sections of the glabrous skin from the hindpaws (30 μm) were cut with a cryostat (Leica CM1900) and thaw-mounted on gelatin-coated slides for processing. Posteriorly, the skin sections were washed in 0.1 M PBS, three times for 10 min each, incubated for 1 h with a blocking solution consisting of 3% Normal Donkey Serum and 0.3% Triton X100 in 0.1 M PBS, and then incubated for 12 h with a PGP-9.5 primary antibody (Protein gene product 1:3,000; Cedarlane; catalog number CL7756AP). Subsequently, preparations were washed in PBS and then incubated for 3 h with the secondary antibody (Cy3 monoclonal donkey anti-rabbit 1:600; Jackson ImmunoResearch; Catalog number 711-165-152). Later, skin sections were washed in PBS, dehydrated through an alcohol gradient (70, 80, 90, and 100%), cleared in xylene, and coverslipped with DPX mounting medium. For the quantification of the intraepidermal nerve fiber density (IENF), initially, at least 10 separate skin sections were scanned at low magnification (10x) to identify the areas with the best integrity thorough an epifluorescence microscope (Axio Scope. A1, Carl Zeiss, Jena, Germany). Then, an image of this area, of a least five different skin sections, was obtained at 20x magnification using a Carl Zeiss scanning confocal laser microscope (model LSM 800, Jena, Germany). The Z-stack images were analyzed using ImageJ software (National Institutes of Health) to determine the total length of nerve fibers. To do this, the nerve fibers innervating the *epidermis* were manually traced using the freehand line tool. The determination of the area of evaluation was obtained by manually tracing the area of the *epidermis*. IENF data are presented as the mean of the total length of nerve fibers (µm) per 100 µm^2^ of the *epidermis* (Pham et al., 2018).

### Statistical Analysis

All data are expressed as mean ± standard error of the mean (SEM). Sample sizes are displayed in the figure legend of each figure. Statistical analyses were performed using GraphPad Prism 8 (GraphPad Software, Inc.). To determine the main effects of treatments (Diet and STZ) and their interaction on variables, Two-Way ANOVA was used. In the case of time as an extra factor contributing to outcome, we used Repeated-Measures Three-Way ANOVA (main effects and interactions) followed by Tukey or Bonferroni post-hoc test. In the case of the time-drive analgesia assessment of drugs in the neuropathic pain induced by cafeteria diet and STZ, we used Repeated Measures Two-Way ANOVA followed by Bonferroni comparison. The area under the curve of the analgesic effect was compared with independent samples *t*-test. From previous pilot studies regarding the 50% withdrawal threshold assay, we had determined that 8–10 animals per group were adequate for an 80% statistical power and alpha cut-off at 0.05. Therefore, all along the study, *p* < 0.05 was considered to indicate a statistically significant difference.

## Results

### Establishment of a T2DM Mouse Model

In this study, female C57BL/6J mice were fed with a CAF-style diet followed by low-doses of STZ to establish a T2DM mouse model. The success of the establishment of T2DM was evaluated by changes in body composition (body weight, BMI, and the amount of visceral adipose tissue), blood glucose levels, OGTT, ITT, and levels of circulating insulin. Changes in body weight during the entire experimental period are illustrated in Figure 1B. At the beginning of the experiment, all the mice exhibited similar body weight (20.9 ± 0.2 g). After 8 weeks of consumption of different diets, body weight was significantly increased in the CAF + VEH (30 ± 0.9 g) and CAF + STZ (31.9 ± 1.6 g) groups compared with the STD + VEH (22 ± 0.5 g) and STD + STZ (22.7 ± 0.6 g) groups. After the STZ or VEH injections, no significant changes in body weight were observed between STD + VEH and STD + STZ mice, which reached a final body weight of 26.2 ± 1.2 g and 26.1 ± 0.8 g, respectively. For the CAF + VEH group, these mice gained weight rapidly and linearly until reaching a final body weight of 52.6 ± 2.5 g. Finally, mice from the CAF + STZ group (final BW 38.9 ± 2.4 g) significantly gained weight in comparison with the mice in the STD + VEH and STD + STZ groups, but weight gain was slower and to a lesser extent than mice from the CAF + VEH group. The statistical analysis showed that the CAF diet was the factor with the highest effect on body weight [F_(1,36)_ = 109.5, *p* < 0.001], the interaction was also statistically significant [F_(1,36)_ = 7.9, *p* < 0.01] in the Three-Way ANOVA. Similarly, at 24th-week post-STZ, the amount of visceral adipose tissue or the BMI from mice of the STD + VEH group were not significantly different compared to those values in mice from the group STD + STZ. In contrast, mice from the CAF + VEH group showed the highest amount of intraperitoneal adipose tissue [F_(1,36)_ = 208.68, *p* < 0.001] and the highest BMI [F_(1,36)_ = 227.17, *p* < 0.001] compared to mice in the STD + VEH and STD + STZ groups. The mice from the CAF + STZ group also showed an increase in intraperitoneal adipose tissue [Interaction CAF × STZ, F_(1,36)_ = 8.8, *p* < 0.01] and BMI [Interaction CAF × STZ, F_(1,36)_ = 15.1, *p* < 0.01] compared to the STD + VEH and STD + CAF mice, but to a lesser extent than the mice from the CAF + VEH group (Supplementary Table S2).

Non-fasting glucose measurements showed that baseline blood glucose levels were 121 ± 3 mg/dL. Mice from the STD + VEH and STD + STZ groups showed blood glucose levels close to 120 mg/dL throughout the entire experimental period. In contrast, mice from the CAF + VEH group had significantly higher glucose levels (162 ± 7.2 mg/dL) compared to those from STD + VEH and STD + STZ groups. Mice from the CAF + STZ group displayed glucose levels similar in magnitude compared to those from the CAF + VEH group until week eight post-STZ injections. However, at week 16 post-STZ injections, the glucose levels in CAF + STZ mice, but not in CAF + VEH mice, were above 200 mg/dL, indicating the establishment of hyperglycemia (Figure 1C). The three-way ANOVA statistical analysis showed that the CAF diet was the factor with the most influence on blood glucose levels [F_(1,36)_ = 74.1], and it was potentiated by STZ after 16 and 24 weeks post-STZ as revealed by the significant interaction at these time points in the statistical analysis.

To identify whether the mice of the different experimental groups had impaired glucose tolerance, an OGTT was performed at 4 or 24 weeks post-STZ injections (Figures 1D–G). In both time points, the results showed that blood glucose levels were maximum at 15 min after the oral load of the glucose solution and returned to baseline values by 60 min (Figures 1D, F) in mice from STD + VEH, STD + STZ, and CAF + VEH groups. In contrast, the glucose levels from the CAF + STZ group were maintained above 300 mg/dL for 30 min after glucose load, and even after 120 min, blood glucose levels were still above 200 mg/dL. The OGTT results were also illustrated with the AUC analysis, where mice from CAF + STZ group showed the highest AUC values at 4 (Figure 1E) and 24 (Figure 1G) weeks post-STZ injections [F_(1,36)_ = 12.17; F_(1,36)_ = 11.73 respectively, *p* < 0.05 for the interaction CAF × STZ, Two-Way ANOVA].

**FIGURE 2:**
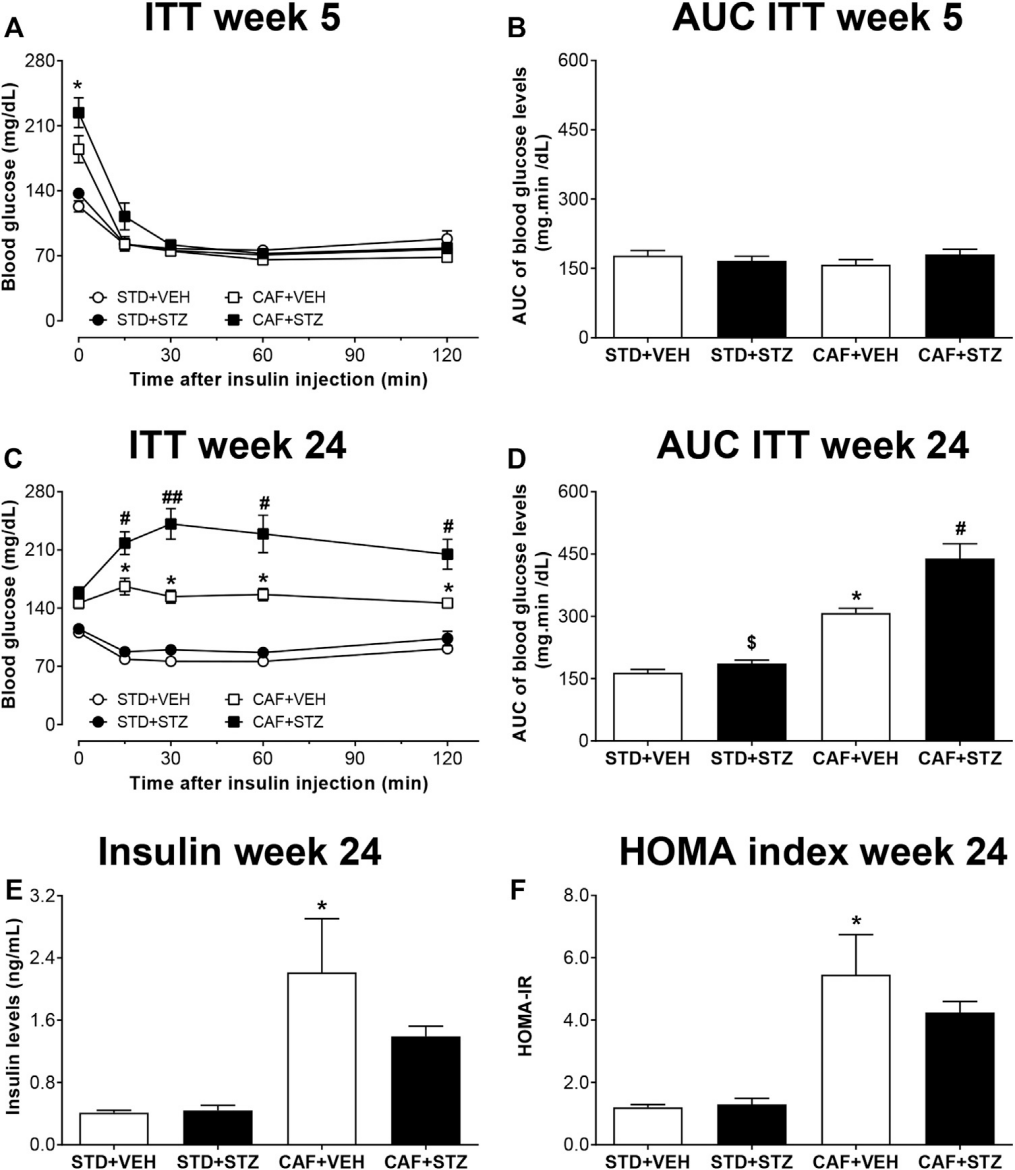
Insulin tolerance test (ITT), insulin levels, and HOMA-index in C57BL/6J female mice with type-2 diabetes induced by CAF diet and low doses of STZ. Panels **(A)** and **(C)** show the blood glucose levels during an ITT at five or 24 weeks after STZ-injections, respectively. Panels **(B)** and **(D)** are showing the area under the curve (AUC) calculated from the ITT. **(E)** Serum insulin levels and **(F)** HOMA2 index calculated 24 weeks post-STZ. Values are means ± SEM (n = 8–10). In **(A)** *p < 0.05 for CAF [F_(1,36)_ = 7.01]. No statistical differences were observed in **(B)**. In **(C)** *p < 0.05 for CAF [F_(1,36)_ = 107.9]; # (*p* < 0.05) ## (*p* < 0.01) for the interaction CAF × STZ [F_(1,36)_ = 5.5]. In **(D)** *p < 0.05 for CAF [F_(1,30)_ = 76.81], $ (*p* < 0.05) for STZ [F_(1,30)_ = 11.55], # (*p* < 0.05) for the interaction CAF × STZ [F_(1,30)_ = 5.9]. In E, *p < 0.05 for CAF [F_(1,25)_ = 30.71]. No statistical differences were observed for STZ or CAF × STZ interaction (*p* = 0.09). In **(F)** *p* < 0.05 for CAF [F_(1,25)_ = 52.82]. No statistical differences were observed for STZ or CAF × STZ interaction. Data in **(A)** and **(C)** were analyzed by Repeated-Measures Three-Way ANOVA followed by Bonferroni multiple comparison. Data in **(B–F)** were analyzed by Two-Way ANOVA. STD: Standard diet; CAF: Cafeteria diet; VEH: Vehicle; STZ: Streptozotocin.

Similarly, to identify whether mice displayed insulin resistance, an ITT was performed 5 or 24 weeks post-STZ injections (Figures 2A–D). At week 5, it was observed that exogenous insulin lowered the blood glucose levels from mice of the four experimental groups (Figures 2A, B). However, at 24 weeks after STZ injections, the ability of insulin to reduce blood glucose was dramatically impaired in the CAF + VEH and CAF + STZ groups (Figure 2C). However, significantly higher insulin resistance was observed in the CAF + STZ group as compared to the CAF + VEH group (Figure 2D). Likewise, it was observed that serum insulin levels were significantly greater in the CAF + VEH (2.2 ± 0.7 ng/ml) and CAF + STZ (1.4 ± 0.1 ng/ml) groups compared to the STD + VEH and STD + STZ groups (both 0.4 ± 0.1 ng/ml) (Figure 2E). Finally, the calculated HOMA-IR values of the mice in CAF + VEH and CAF + STZ groups were significantly higher compared to the STD + VEH and STD + STZ groups (Figure 2F).

### Mice With T2DM Developed Long-Lasting Hindpaw Mechanical Allodynia

**FIGURE 3:**
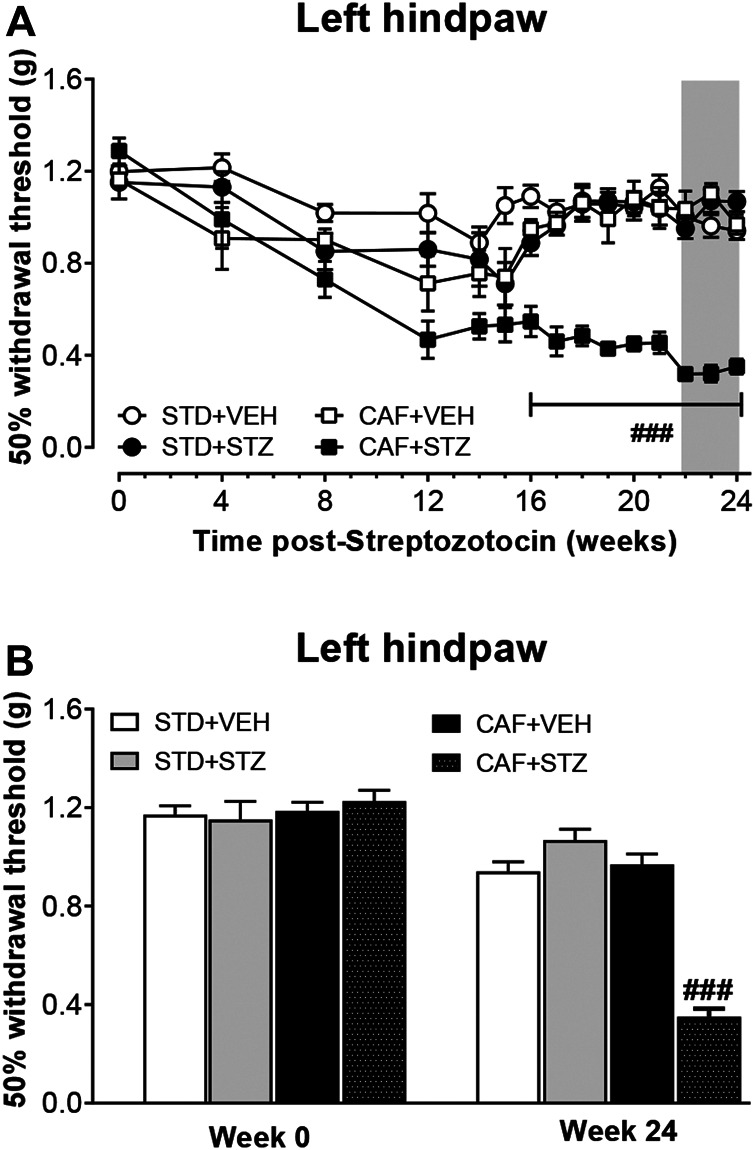
Development of mechanical skin hypersensitivity in female mice with T2DM. **(A)** is showing the time course of the development of hindpaw mechanical hypersensitivity. **(B)** is showing the 50% withdrawal threshold values before STZ injection and 24 weeks-post STZ, respectively. Values are mean ± SEM (n = 10). In **(A)**, ###*p* < 0.001 for the interaction CAF × STZ [F_(1, 36)_ = 42.4]. In **(B)** ###*p* < 0.001 for the interaction CAF × STZ [F_(1, 36)_ = 86.6]. Data in **(A)** were analyzed by Repeated-Measures Three-Way ANOVA followed by Bonferroni multiple comparison. Data in **(B)** were analyzed by Two-Way ANOVA. STD: Standard diet; CAF: Cafeteria diet; VEH: vehicle; STZ: streptozotocin. The gray rectangle is indicating the period in which the analgesic drugs were tested.

To determine whether diabetic mice developed tactile allodynia as a sign of painful DPN, 50% withdrawal thresholds were measured. Before the STZ injections, no significant 50% withdrawal threshold differences were found among the four experimental groups. In contrast, after STZ injections, only mice from CAF + STZ group developed a slow and progressive mechanical hypersensitivity in the left hindpaw. This mechanical hypersensitivity was characterized by a significant reduction of 50% withdrawal threshold (PWT) that was detected as early as week 12 and maintained until week 24 post-STZ injections (Figure 3A) The statistical analysis revealed an important effect of the interaction between CAF diet and STZ [F_(1,36)_ = 42.4] for overall experiment. During weeks 22–24 post-STZ, the reduction on PWT reached values of 0.35 ± 0.03 g for the left hindpaw. In contrast, no statistically significant changes in mechanical sensitivity from mice of the STD + VEH, STD + STZ, or CAF + VEH were observed at week 24 compared to their respective values before STZ-injections (Figure 3B).

### Evaluation of Different Neuropathic Pain Drugs and Diclofenac on Mechanical Allodynia

**FIGURE 4:**
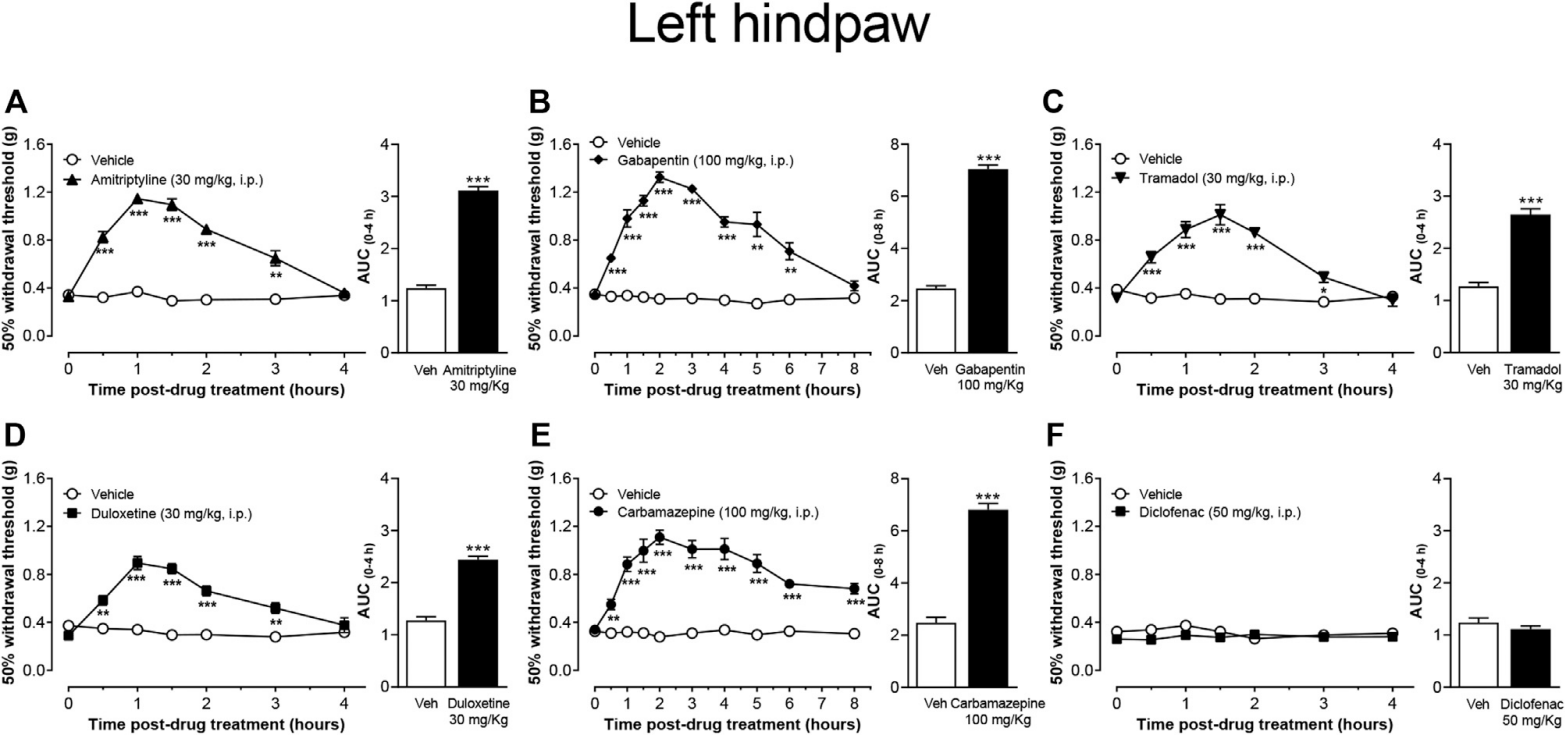
Effect of the most common drugs used in the treatment of neuropathic pain, and diclofenac on mechanical skin hypersensitivity on the left hindpaw of CAF + STZ mice. Values are means ± SEM (n = 8). **p < 0.01, ***p < 0.001; significantly different from the vehicle group by Repeated Measures Two-Way ANOVA, followed by Bonferroni’s test. Attached the AUC of the effect is also shown, ***p < 0.001, *t*-student test.

In this study, the effect of different drugs used as first-line to treat diabetic DPN in human was evaluated on T2DM-induced tactile allodynia. The results showed that the 50% withdrawal threshold of both hindpaws was significantly increased in diabetic mice that received one i.p. injection of amitriptyline (Figure 4A), gabapentin (Figure 4B), tramadol (Figure 4C), duloxetine (Figure 4D), and carbamazepine (Figure 4E). The anti-allodynic effect started 30 min after the treatment injection of all drugs. This pharmacological effect lasted at least 2.5 h in the mice treated with amitriptyline, duloxetine, or tramadol, 5.5 h in the mice treated with gabapentin, and at least 7 h in the group treated with carbamazepine. In contrast, diclofenac did not affect the 50% withdrawal threshold values of either hindpaw up to 4 h post-injection compared with either the pre-injection withdrawal threshold or with vehicle-treated animals at the corresponding time points. (Figure 4F).

### T2DM Mice Showed a Reduction of Intraepidermal Nerve Fibers in the Glabrous Skin of the Hindpaw

**FIGURE 5:**
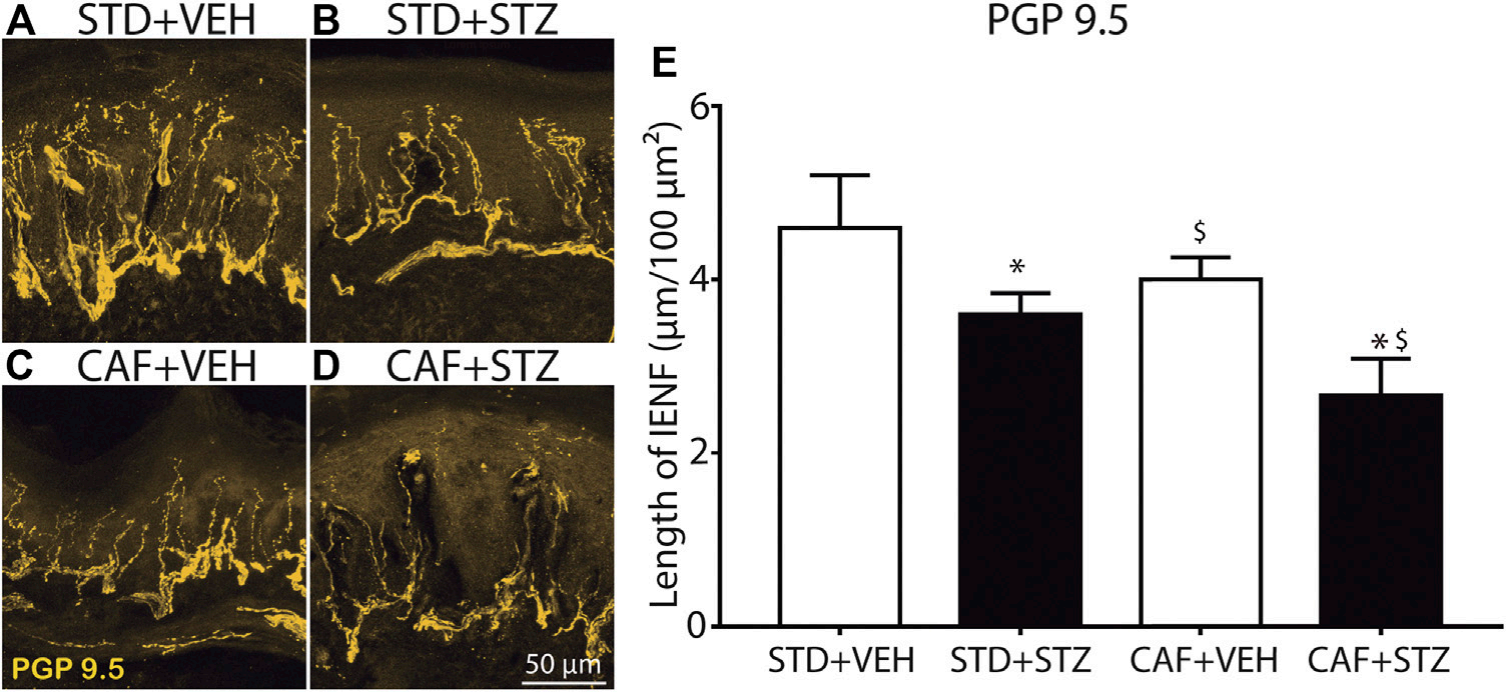
Loss of epidermal nerve fibers in footpads of female mice with diabetes induced by cafeteria diet and low doses of STZ. Representative confocal images of intraepidermal PGP-9.5 immunopositive nerve fibers from the glabrous skin of the hindpaw, obtained from STD + VEH mice **(A)** STD + STZ mice **(B)** CAF + VEH **(C)**, and CAF + STZ **(D)**. **(E)** Quantification of the IENF in each experimental condition. Values are means ± SEM (n = 6). *p < 0.05 for CAF [F_(1,18)_ = 5.45], $ (*p* < 0.05) for STZ [F_(1,18)_ = 9.14], the interaction CAF × STZ did not reach statistical significance, * and $ on the CAF × STZ column represent the separate effect of each factor on the PGP-9.5 nerve length. Two-Way ANOVA.

To evaluate the IENF density in skin sections from the hindpaw, skin sections were processed by IHC and labeled with a primary antibody against PGP-9.5 (a pan-neuronal marker). Quantitative analysis of the density of PGP-9.5 immunopositive nerve fibers axons revealed that mice from the STD + STZ and CAF + VEH groups had reduced values of length of IENF compared to the control group (STD + VEH) as evaluated in the Two-Way ANOVA. Mice from the CAF + STZ group showed the loss of density of PGP-9.5 immunoreactive axons innervating the *epidermis* from the hindpaw skin as the arithmetical sum of the effects elicited by CAF and STZ non-implying potentiation, however, the effect observed was not negligent from a biological point of view, as it concerns a nerve terminal (Figure 5).

## Discussion

### The Combination CAF Diet Plus Multiple Low-Doses of STZ Induced T2DM in Mice

T2DM is the most common type of diabetes and is often associated with obesity. This disease is increasing in pandemic proportions largely driven by a sedentary living and high-energy dietary intakes (Kolb and Martin, 2017; IDF, 2019). Thus, there is an urgent need to develop effective mechanism-based therapies to treat this disease and/or its complications. Preclinical models of T2DM play a pivotal role for the study of its pathophysiology and complications, as well as the rational screening of effective anti-diabetic drugs (Sullivan et al., 2008; Islam and Wilson, 2012; King, 2012; Kleinert et al., 2018). However, translation of findings from these models to human is often challenging due to most likely that many of the current preclinical models of T2DM do not appear to reproduce the main clinical features of patients and/or these models are relatively expensive and not easily available for a large number of researchers (King, 2012; Kleinert et al., 2018). This study reports, for the first time, the characterization of a translational non-genetic mouse model of painful DPN, in which T2DM was induced by a CAF diet followed by low-doses of STZ. The CAF diet was chosen because it closely mimics the unhealthy diet common to humans, which in turn contributes to weight gain and obesity, two frequent features of T2DM (Sclafani and Springer, 1976; Sampey et al., 2011). However, high-fat diets, such as the CAF diet, alone does not lead to diabetes in C57BL/6J mice (Kleinert et al., 2018). Thus, β-cell impairment was induced by the injection of four low-doses of STZ. This model in female C57BL/6J mice resulted in changes in body composition, evidenced by significant weight gain, and an increase in BMI and the amount of visceral adipose tissue, long-term hyperglycemia, hyperinsulinemia, resistance to insulin, and impaired glucose tolerance. Additionally, CAF + STZ mice slowly developed mechanical pain hypersensitivity and showed a significant reduction of epidermal PGP -9.5^+^ nerve fibers of the hindpaw skin. Altogether, this reverse translational study suggests that this model of painful DPN is easily accessible, fairly economical, and with a high success rate that closely parallels the common course of the human disease where obesity and β-cell dysfunction contribute to the development of T2DM.

Our results showed that CAF-diet fed mice exhibited rapid and significant weight gain. This is consistent with the results of several studies showing that an increase in dietary fat and carbohydrate content has been shown to produce obesity in various strains of mice and rats (Collins et al., 2004; Sampey et al., 2011). However, paralleling the human T2DM where weight loss can occur in patients with longstanding disease (Yang et al., 2016), CAF-diet fed mice who received low doses of STZ also experienced eventual weight loss.

Hyperglycemia is one of the main features of diabetes and determination of blood glucose level is widely used as a marker for diagnosis and disease progression (Stolar, 2010; ADA, 2019). In this study, mice with the CAF diet + STZ showed non-fasting blood glucose levels above 200 mg/dL from week 16 until the end of the study. These mice also displayed a glucose-intolerant phenotype at 4, and 24 weeks after STZ injections the combination potentiated the effects in term of body weight, blood glucose, and insulin resistance as is evident by blood glucose levels above 200 mg/dL even at 120 min after a glucose load during an OGTT. Our results also showed that CAF + STZ mice developed insulin resistance only at 24 weeks post-STZ injections, as revealed by the potentiated interaction (CAF × STZ) in the statistical analysis. In contrast, mice with CAF + VEH showed glucose levels closer to 150 mg/dL, a modest impairment in glucose tolerance, and slight insulin resistance and hyperinsulinemia, features of a prediabetic state. These results agree with human data showing that not all obese patients develop diabetes (Ogden et al., 2015; Malone and Hansen, 2019). Altogether these results showed that the consumption of a cafeteria diet for long periods in conjunction with dysfunction of β-pancreatic cells leads to the development of T2DM. To our knowledge, we report for the first time the validation and characterization of T2DM using CAF diet along with low-doses of STZ in mice. However, we should recognize that previous studies performed in rodents have demonstrated that the combination of low-doses of STZ with a high-fat diet can recapitulate certain features of diet-induced T2DM seen in humans (Srinivasan et al., 2005; Zhang et al., 2008; Nath et al., 2017). However, it has been shown that the CAF diet is a more robust model of human metabolic syndrome compared to high-fat diet both in rats and mice (Sampey et al., 2011; Higa et al., 2014). Finally, another advantage of using the CAF diet is that this diet more accurately reflects the variety of inexpensive highly palatable, energy-dense foods that is easily available in Western society and associated with the current obesity epidemic (West and Kalbfleisch, 1971; Sclafani and Springer, 1976; Sampey et al., 2011).

### Diabetic Mice Slowly Developed Mechanical Allodynia

Nociceptors of the dorsal root ganglia and dorsal horn can become hyperexcitable in response to pathological conditions such as diabetes, which in turn may lead to the development of painful DPN (Todorovic, 2016). DPN is one of the most common and disabling complications with 30–50% of T2DM patients developing PDN (Candrilli et al., 2007; Singh et al., 2014; Juster-Switlyk and Smith, 2016). It commonly presents with progressive distal dysesthesias, pain, and/or sensory loss. Histologically, DPN is associated with loss of the nerve fiber axons within the skin (Rosenberger et al., 2020). In this study, we found that mice with CAF diet plus STZ treatment, but not mice with CAF diet or STZ treatment given alongside a normal diet, developed long-term mechanical allodynia in both hindpaws, which was detected as early as 12 weeks post-STZ treatment. Our study agrees with a previous report that the CAF diet alone given for 12 weeks does not alter the tactile sensitivity assessed by von-Frey monofilaments in rats (Hossain et al., 2020). However, our results differ from other studies in that mechanical allodynia has been reported in mice after STZ treatment alone (Wright et al., 2004; Christianson et al., 2007; Johnson et al., 2008; McGuire et al., 2009; Urban et al., 2010). While the reasons behind these contrasting results are unknown, it possible to suggest that the dose of STZ used by others (180–210 mg/kg, which usually induced T1DM, vs. four consecutive injections of 30 mg/kg in our study) and method of behavioral evaluation partially explain these behavioral differences. It is worthy to mention that this study reports for the first time a longitudinal assessment of neuropathic pain-like behaviors in mice with CAF diet plus STZ treatment. This longitudinal approach revealed that the mechanical allodynia was not evident at early time points of the disease stages. Our result is consistent with previous studies in which the development of mechanical allodynia was evident only at late stages in rats with T2DM induced by high-fat/high-fructose diet and low-doses of STZ (Barriere et al., 2018) and/or with humans studies showing damage to peripheral nerves at late stages of the disease (Said, 2007).

### Mechanical Allodynia Induced by T2DM is Decreased by the Conventional Drugs Used to Treat Neuropathic Pain in Humans.

One goal of this study was to validate a mouse model of painful DPN that not only would closely mirrors the natural history of the human disease but also a reliable model that allows a rational screening of new analgesic drugs to treat painful DPN. In the present study, five different analgesics significantly decreased mechanical allodynia induced by T2DM in mice. These findings agree with clinical observations as these drugs have been included in the guidelines by the American Diabetes Association and European Federation of Neurological Societies Task Force to treat painful neuropathy in diabetic patients (Attal et al., 2010; Pop-Busui et al., 2017; Iqbal et al., 2018). Conversely, we found that acute administration of a clinically relevant dose of diclofenac lacked anti-allodynic effect. This result agrees with previous observations where diclofenac has no efficacy in treating painful diabetic neuropathy in humans (Tinoco-Samos et al., 2013) or mechanical allodynia in rats with STZ-induced T1DM (Yamamoto et al., 2009). Finally, this model provides a long window of evaluation time (at least 8 weeks) which might allow the analgesic evaluation of the different drugs given acutely, using the minimum number of experimental animals, as long as there is enough time for a washout period of at least seven drug-half-lives. Moreover, it also offers the possibility of evaluating chronic treatments in order to determine if the effect is maintained for long periods of time or to identify drug-related negative side effects. These results together suggest that this model has translational utility due to it may represent a reliable platform for predicting the clinical efficacy of drugs to treat painful DPN associated with T2DM.

### Mice Fed with CAF Diet and Injected with Multiple Low-Doses of STZ Showed a Reduced IENF Density

Intraepidermal nerve fibers (IENF) are directly associated with functional innervation of the skin, and the reduction of IENF density constitutes one of the main histological features in T2DM patients (Ekman et al., 2020). In fact, quantification of epidermal innervation is considered as a reliable and minimally invasive methods of diagnosing and staging diabetic neuropathy (Beiswenger et al., 2008). In the current study, we observed that STZ treatment by itself caused a significant reduction in epidermal PGP -9.5^+^ nerve fibers in agreement with other studies (Stavniichuk et al., 2014; Rojas et al., 2019). Animals receiving long-term CAF diet also showed a discrete, but significant effect on PGP -9.5^+^ nerve fibers. The combination of CAF diet with STZ treatment was observed as the arithmetical sum of both factors by themselves on the density of epidermal PGP -9.5^+^ nerve fibers, but without observing statistical potentiation (in the Two-Way ANOVA), thus suggesting that both factors could be acting by a similar mechanism non-implying potentiation, however, the effect observed was not negligent from a biological point of view, as it concerns a nerve sensitive terminal.

### Limitations

The present study has some limitations. Firstly, just female mice were used in this study. However, due that T2DM has a similar prevalence in female and male patients (IDF, 2019), future studies are needed to reproduce these results in male mice. Secondly, we have found that there is a reduction PGP -9.5-immunoreactive intraepidermal of nerve fibers in the hindpaw skin of mice with T2DM which is consistent with the loss of cutaneous innervation in human patients with diabetes (Kennedy et al., 1996; Herrmann et al., 1999; Christianson et al., 2003; Christianson et al., 2007). However, we should recognize that this parameter was determined only at 6 months after STZ injections. It is possible that the reduction of cutaneous innervation may occurs at earlier time points as it has been reported in models of T1DM (Christianson et al., 2003; Christianson et al., 2007; Johnson et al., 2008). Thirdly, while we used PGP -9.5 as widely pan-neuronal marker, this antibody does not allow to distinguish what subtype of C-fiber nociceptors (peptidergic vs. non-peptidergic) are predominantly affected in the present T2DM model. Given that it has been shown that peptidergic and non-peptidergic nociceptive neurons are differentially damaged under T1DM (Johnson et al., 2008), future studies are needed to identify the biochemical phenotype of nerve fibers affected in this model of T2DM. Fourthly, we have characterized the time-course of the development of mechanical allodynia in this T2DM as a neuropathy sign. While this behavioral endpoint is widely used as a surrogate of pain in humans with diabetic peripheral neuropathy (Perkins et al., 2001), further studies are needed to evaluate other pain-like behaviors that would allow determining the presence of ongoing pain or sensory deficits in this model.

In conclusion, we have shown that the CAF diet along with low-doses of STZ in mice may be effectively used to generate an easily accessible, fairly economical, and reliable model of peripheral neuropathy associated with T2DM after inducement of obesity and β-cell impairment in female C57BL/6J mice. This model mimics some of the metabolic features, mechanical allodynia, and a reduction of cutaneous innervation reported in humans with T2DM. Thus, this preclinical mouse model could be a useful and translational tool in evaluating the efficacy of new drugs to treat painful diabetic neuropathy and improve translation.

## Data Availability

The raw data supporting the conclusions of this article will be made available by the authors, without undue reservation.
