# Supplementary material for: Characterization of Mechanical Allodynia and Skin Innervation in a Mouse Model of Type-2 Diabetes Induced by Cafeteria-Style Diet and Low-Doses of Streptozotocin
Source: Front Pharmacol. 2021 Feb 3;11:628438. doi: 10.3389/fphar.2020.628438 (PMC7957928; doi:10.3389/fphar.2020.628438)
Supplement: Supplementary file 1 [file datasheet1.docx]

Supplementary Material

# Supplementary Table 1. Menu, CAF-diet food items, and dietary composition.

| **Day of week** | **Food**  **Menu per day** | **Amount for a cage with 4 mice (g)** | **Total Kcal** | **Total diet Kcal/ day /mice** | **Macro**  **Nutrients per day menu** | **Total Energy Value** |
| --- | --- | --- | --- | --- | --- | --- |
| **MONDAY** | **Turkey ham** | 6.7 | 8.4 | **36.9** | **Fat** | 27.9% |
|  | **Milk chocolate bars** | 4.9 | 25.1 |  |  |  |
|  | **Cheese chips** | 6.7 | 35.1 |  | **Carbs** | 57.3% |
|  | **Rodent Diet 5001** | 5.8 | 27.1 |  |  |  |
|  | **Marshmallow** | 12.4 | 41.5 |  |  |  |
|  | **Orange-flavored soft drink** | 50 mL | 10.5 |  | **Protein** | 14.8% |
|  | **Water** | 50 mL | 0.0 |  |  |  |
| **TUESDAY** | **Chocolate Cookie** | 11.3 | 53.6 | **46.2** | **Fat** | 44.0% |
|  | **Potato Chips** | 6.5 | 35.1 |  |  |  |
|  | **Cheese chip** | 6.7 | 35.1 |  | **Carbs** | 27.4% |
|  | **Rodent Diet 5001** | 5.8 | 27.1 |  |  |  |
|  | **Turkey Sausage** | 13.5 | 23.2 |  | **Protein** | 28.5% |
|  | **Orange-flavored soft drink** | 50 mL | 10.5 |  |  |  |
|  | **Water** | 50 mL | 0.0 |  |  |  |
| **WEDNESDAY** | **Turkey ham** | 6.7 | 8.4 | **33.2** | **Fat** | 45.1% |
|  | **Hazelnut Spread** | 9.2 | 51.9 |  |  |  |
|  | **Cheese chips** | 6.7 | 35.1 |  | **Carbs** | 38.7% |
|  | **Rodent Diet 5001** | 5.8 | 27.1 |  |  |  |
|  | **Orange-flavored soft drink** | 50 mL | 10.5 |  | **Protein** | 16.1% |
|  | **Water** | 50 mL | 0.0 |  |  |  |
| **THURSDAY** | **Turkey ham** | 6.7 | 8.4 | **18.9** | **Fat** | 29.2% |
|  | **Milk chocolate bars** | 4.9 | 25.1 |  |  |  |
|  | **Rodent Diet 5001** | 5.8 | 27.1 |  | **Carbs** | 46.5% |
|  | **Cola soft drink** | 50 mL | 10.5 |  |  |  |
|  | **Water** | 50 mL | 0.0 |  | **Protein** | 24.9% |
| **FRIDAY, SATURDAY AND SUNDAY** | **Chocolate Cookie** | 11.3 | 53.6 | **48.9** | **Fat** | 31.7% |
|  | **Rodent Diet 5001** | 5.8 | 27.1 |  |  |  |
|  | **Potato Chip** | 6.5 | 35.1 |  | **Carbs** | 41.3% |
|  | **Turkey Sausage** | 13.5 | 23.2 |  |  |  |
|  | **Marshmallow** | 12.4 | 41.5 |  |  |  |
|  | **Cola soft drink** | 50 mL | 10.5 |  | **Protein** | 27.0% |
|  | **Water** | 50 mL | 0.0 |  |  |  |

# Supplementary Table 2. BMI and weight of visceral adipose tissue 24 weeks post-STZ.

| **PARAMETER** | **SDT+VEH** | **STD+STZ** | **CAF+VEH** | **CAF+STZ** |
| --- | --- | --- | --- | --- |
| BMI (kg/m^2^) | 3.51 ± 0.05 | 3.61 ± 0.05 | 4.83 ± 0.11^***^ | 4.37 ± 0.05^##^ |
| Weight of visceral adipose tissue (g) | 1.72 ± 0.33 | 1.56 ± 0.12 | 7.55 ± 0.49^***^ | 5.67 ± 0.34^##^ |

Values are mean ± SEM. *** P < 0.001 for CAF (BMI, F=227; Adipose tissue F=208.68).

## P < 0.01 for the interaction CAF x STZ (BMI, F=15.1; Adipose tissue F=8.8).
